# Nursing attitudes towards continuous capnographic monitoring of floor patients

**DOI:** 10.1136/bmjoq-2018-000416

**Published:** 2018-09-15

**Authors:** Catherine L Clark, Liza M Weavind, Sara E Nelson, Jennifer L Wilkie, Joel T Conway, Robert E Freundlich

**Affiliations:** 1 Department of Anesthesiology, Vanderbilt University Medical Center, Nashville, Tennessee, USA; 2 Biomedical Informatics, Vanderbilt University Medical Center, Nashville, Tennessee, USA

**Keywords:** adverse events, epidemiology and detection, attitudes, cognitive biases, continuing education, continuing professional development, hospital medicine

## Abstract

**Introduction:**

Nurses’ perceptions of the utility of capnography monitoring are inconsistent in previous studies. We sought to outline the limitations of a uniform education effort in bringing about consistent views of capnography among nurses.

**Methods:**

A survey was administered to 22 nurses in three subacute care floors participating in a pragmatic clinical trial employing capnography monitoring in a large, urban tertiary care hospital. A 5-point Likert scale was used to assess the value and acceptance nurses ascribed to the practice. Means and SD were calculated for each response.

**Results:**

Survey results indicated inconsistency in the valuation of capnography, coupled with varying degrees of acceptance of its use. The mean for the level of perceived impact of capnography use on patient safety was 3.86, yet the perceived risk of removing capnography was represented by a mean of 2.57. The levels of urgency attached to apnoea alarms (mean 3.57, SD 1.57) were lower than those for alarms for oxygen saturation violations (mean 3.67, SD 1.32). The necessity for pulse oximetry monitoring was perceived as much higher than that for capnography monitoring (mean 1.76, SD 1.34), where ‘1’ represented pulse oximetry as more necessary and ‘5’ represented capnography as more necessary.

**Conclusions:**

Nursing acceptance of capnography monitoring is a difficult endpoint to achieve. There is a need for better accounting for the external and internal influences on nurse perceptions and values to have greater success with the implementation of similar monitoring.

## Introduction

Monitoring end-tidal carbon dioxide concentrations, as a measure of adequate ventilation, has become standard of care in a growing number of high-acuity hospital settings.^1 2^ As the value of capnography monitoring has become apparent to a growing number of clinicians, its employment has gradually expanded beyond the acute care and operating room settings to lower acuity units of the hospital, where nurses may be expected to add this new form of monitoring to their expanding list of duties.^3^ Due to perceived deficits in existing monitoring strategies for low-acuity inpatients,^3–6^ capnography is increasingly being implemented in non-traditional clinical areas. As such, the attitude of non-acute care nurses towards capnography is of increasing importance.

Wide variations among medical staff have been shown to exist in the understanding and acceptance of capnography.^7^ Many healthcare professionals lack an appreciation for the function and value of capnography, despite evidence of its utility as an indicator of respiratory distress.^8^

Influences on attitudes towards capnography may include education, or knowledge translation, as a principal category; other influences include environment and experiences.^7^ Research on technology implementation supports the legitimacy of these categories as barriers to implementation of other new practices and technology.^9–11^ Alternative elements that may also impact attitudes towards capnography are cognitive styles and professional values.^9 12 13^

We are unaware of research that has examined nurse attitudes towards capnography in terms of the combination of these exact classifications; however, a qualitative evaluation of capnography use did report findings that revealed generally positive attitudes of nurses towards capnography after a fixed baseline education.^14^ So-called ‘buy-in’ has been shown to be a helpful precondition for implementation of organisational initiatives, and efforts to better understand how to optimise ‘buy-in’ are an area of active research.^15^

The perceptions of users towards new technology are critical factors in its acceptance and successful implementation.^9^ Over the course of a separate study examining the impact of continuous capnography monitoring for low-acuity inpatients, we sought to collect feedback informally from nurses taking care of enrolled patients to better understand nurses’ attitudes towards capnography monitoring. Based on our preliminary conversations, we hypothesised that nurses would demonstrate inconsistency in their valuation and acceptance of capnography, despite being exposed to thorough and standardised education on the rationale for its implementation.

## Methods

A survey was prospectively designed to assess nurse attitudes towards the use of capnography monitoring. Staff who chose to participate in the survey were involved in a separate study of continuous capnography monitoring of low-acuity inpatients in which nurses were asked to connect patients to a capnography and pulse oximetry monitor on admission to their unit and respond to notifications for apnoea, heart and respiratory rate violations, as well as low oxygen saturation levels. Two years prior to the initiation of the capnography study, the device manufacturer conducted training on the units where the monitors were introduced, followed by an online training opportunity. When nurses encountered obstacles accessing this material, a nurse educator developed an in-house training module for all nurses to complete. Over the intervening 2 years, continuous efforts were made to ensure ongoing education. The device manufacturer visited participating units again 2–6 months prior to the study. Finally, nurse educators on two floors instructed the staff at their regular meeting, and one educator addressed the capnography study twice. Two to 8 weeks prior to the onset of the capnography monitoring study, educators sent a notice by email to floor nurses; one of the educators representing the floors being monitored sent a second email. At least one educator sent a follow-up email clarifying the silencing of alarms. Routine visits were made by one of the physician investigators (LMW) to remind staff of the function and parameters of the study. New staff were educated using online learning modules.

The survey instrument was administered by two clinical researchers (JTC and CLC) to 22 nurses (20% of 109 nurses on staff) on three different units, including respondents from day and night shifts on each unit. Nurses on multiple midweek shifts for three consecutive weeks (10 May 2017–31 May 2017) were solicited for the survey during breaks from their duties, and 100% of nurses on shift agreed to participate.

The instrument consisted of 18 questions (table 1) examining the ways that the monitoring affected the workflow and perceptions of caregivers. We used the EQUATOR reporting checklist from ‘Good practice in the conduct and reporting of survey research’ (International Journal for Quality in Health Care 15(3)) as a guideline for our paper, as our results were quantitative. The survey sought to indicate the degree to which nurses understand and value capnography monitoring, as well as the influences on their perceptions. In addition, the instrument was designed to compare attitudes towards pulse oximetry versus capnography. A 5-point Likert scale was used to determine the level of positive or negative feelings associated with the use of capnography and its perceived value, with higher scores representing more positive attitudes. The importance that capnography alarms hold for nurses and their comparative value with pulse oximetry alarms were also assessed. A Cronbach’s alpha coefficient was calculated to evaluate the survey’s internal consistency. Student’s t-test was used to compare mean values and a p value of <0.05 was considered statistically significant. Statistical analyses were performed using R V.3.4.3 (R Foundation for Statistical Computing, Vienna, Austria).

**Table 1:** A 5-point Likert scale was used to determine the level of positive or negative feelings associated with the use of capnography and its perceived value, with higher scores representing more positive attitudes

Survey questions:	Mean	SD
Q1. How is your ability to carry out your duties affected by adding capnography monitoring to patient care?	3.00	1.00
Q2. How do you feel patient safety is being affected by capnography monitoring?	3.86	0.65
Q3. How many experiences have you had with patients who had escalations of care that might have been prevented using capnography?	2.05	1.02
Q4. What effect do you think capnography monitoring has on patient satisfaction?	2.38	1.16
Q5. What level of cooperation would you describe patients as having with wearing the capnography cannula?	2.52	0.81
Q6. Which choice best describes your attitude towards the necessity of pulse oximetry monitoring compared with capnography?	1.76	1.34
Q7. Please describe your style of patient communication: direct (telling patients protocols they will follow) or indirect (suggestion that they follow certain protocols).	3.76	0.89
Q8. If capnography monitoring were removed today, do you think patient safety would be more at risk?	2.57	0.51
Q9. Capnography provides important feedback DURING surgery.	3.90	1.00
Q10. Capnography provides important feedback up to 1 hour postsurgery.	3.81	0.98
Q11. Capnography provides important feedback up to 8 hours postsurgery.	3.86	1.01
Q12. Capnography provides important feedback up to 24 hours postsurgery.	3.24	0.94
Q13. Capnography provides important feedback in the presence of certain comorbidities.	3.86	1.01
Q14. Capnography provides important clinical data in the unstable patient.	4.33	1.02
Q15. What level of urgency do you currently assign to an alarm for apnoea?	3.57	1.57
Q16. What level of urgency do you currently assign to an alarm for a respiratory rate violation?	3.38	1.20
Q17. What level of urgency do you currently assign to an alarm for a heart rate violation?	3.57	0.93
Q18. What level of urgency do you currently assign to an alarm for a oxygen saturation (SpO_2_) violation?	3.67	1.32

## Results

**Figure 1:**
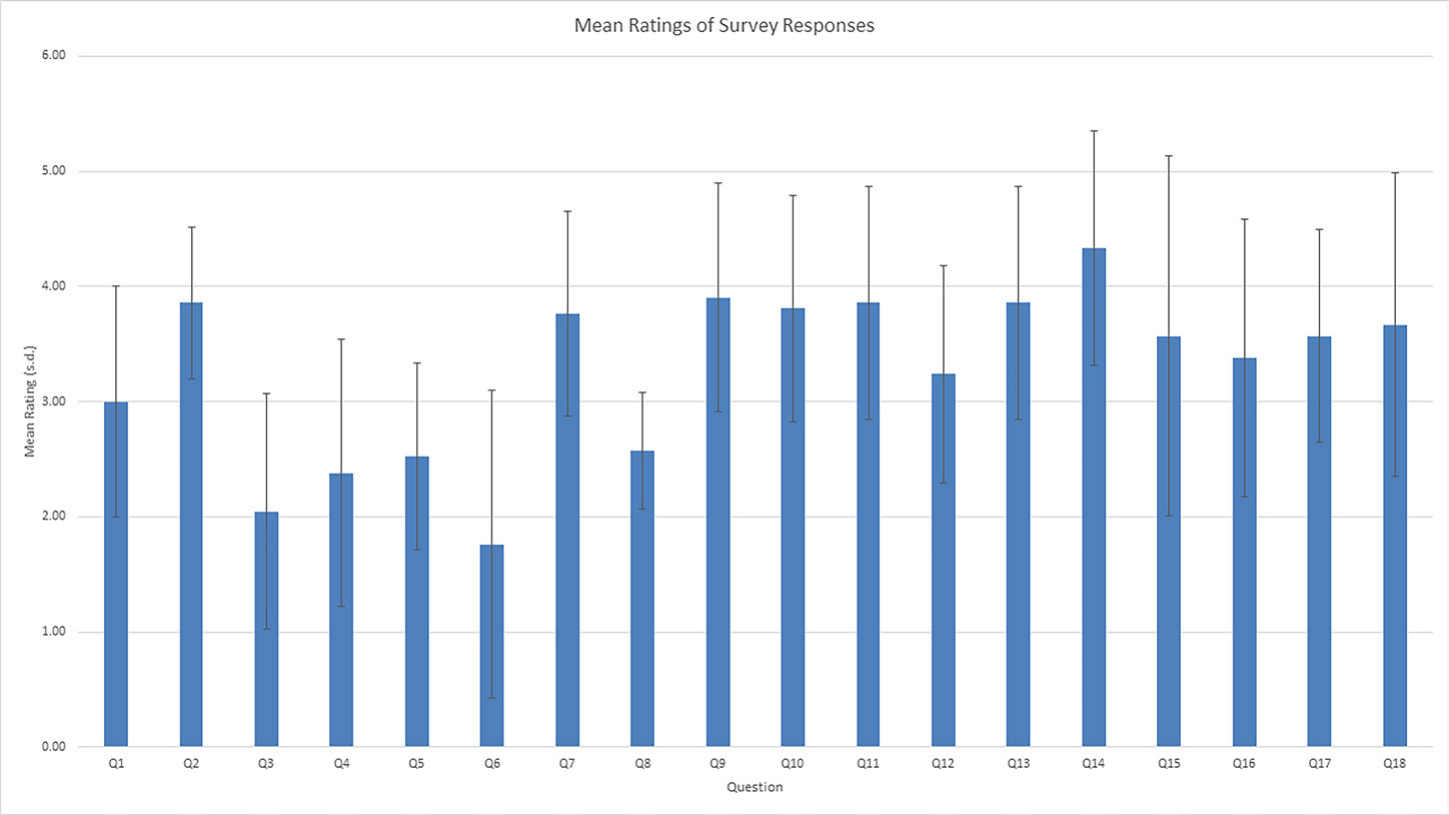
Mean ratings of survey responses. A 5-point Likert scale was used to determine the level of positive or negative feelings associated with the use of capnography and its perceived value, with higher scores representing more positive attitudes.

Analysis of the survey’s internal consistency revealed a Cronbach’s alpha of 0.79. As we hypothesised, participants expressed an inconsistent valuation of the use of capnography (figure 1). While 17 out of 22 respondents (77%) indicated their belief that patient safety was somewhat to very positively affected by capnography monitoring, only 9 of the 22 (41%) surveyed felt patient safety would be more at risk if capnography monitoring were removed today. Twelve nurses (55%) had experienced a few escalations of care that might have been prevented had capnography monitoring been used; none of them had experienced what they characterised as ‘many’ escalations of care that might have been averted with capnography monitoring. Ten nurses (45%) had not ever been part of an escalation that they believe would have had a different outcome with capnography monitoring.

Perceived importance of capnography data was measured at varying time-points (during surgery, 1 hour postsurgery, 8 hours postsurgery and 24 hours postsurgery). After calculating the mean Likert score, we found that capnography monitoring was most highly valued 8 hours after surgery, with 17 nurses (77%) somewhat or strongly agreeing that the feedback was important at that time-point, compared with 1 hour postsurgery, with 12 (55%) somewhat or strongly agreeing, followed by 24 hours postsurgery, with 10 (45%) somewhat or strongly agreeing.

The mean of the Likert scores measuring perceived value of capnography on a 5-point scale was 3.47. Postoperative context, such as stability of the patient, was strongly correlated to nursing attitudes towards capnography; nursing attitudes comparing capnography to pulse oximetry were similarly strongly correlated to postoperative context. The effect that context, such as stability of the patient, had on nurse attitudes was seen clearly, as were the results when capnography was compared with pulse oximetry.

Questions regarding the level of importance that nurses attributed to alarms associated with capnography showed that apnoea alarms signified extreme urgency or no urgency more often than medium levels of urgency, whereas alarms for heart rate violations had more reactions of midlevel urgency, with fewer nurses feeling those alarms were extremely urgent or not at all urgent. The level of perceived urgency that nurses assigned to apnoea alarms resulting from capnography monitoring (mean=3.57, SD=1.57) was similar to the level of perceived urgency that nurses assigned to low oxygen saturation violation alarms resulting from pulse oximetry monitoring (mean=3.67, SD=1.32). Comparing the responses of these two questions concerning perceived urgency was insignificant, returning a p value of 0.833. (See online supplementary appendix for survey questions and options for responses.)

10.1136/bmjoq-2018-000416.supp1 Supplementary file 1

## Discussion

Responses to our survey showed wide variance in levels of acceptance of the value of capnography monitoring. While most participants agreed that capnography monitoring improves patient safety and provides important feedback following surgery, answers to several questions on the survey indicated that nurses did not see its feedback as valuable, especially compared with that of pulse oximetry. Most respondents felt that patients would not be more at risk if capnography were removed today, yet they assigned apnoea alarms the highest number of ‘extremely urgent’ scores compared with other alarms.

The variation in valuation of capnography monitoring we observed in this study underscores the challenges inherent to adopting new technologies. A conceptual framework that underpins our survey questions, and which systematically addresses these challenges, is the Diffusion of Innovation Theory. This theory outlines several predictors of the adoption rate of technologies; their relative advantage, which our study examined closely, is of essential importance.^16^ The survey we administered targeted perceived relative advantage from several different vantage points by inquiring about perceived risk without capnography, perceived value of feedback at varying intervals postsurgery, in the presence of certain comorbidities, and in unstable patients.

Barriers that can be foreseen and mitigated, according to Diffusion of Innovation Theory, such as lack of compatibility with current organisational environment and culture, as well as complexity, affect the level of need for education. Both barriers are used as a template for success in our articulation of implementing capnography monitoring.^16^ Perceived value, rather than actual value, is a determinant of adoption,^11^ and the perceptions and attitudes of nurses drive patient care significantly, which underscores the importance of understanding the attitudes we measured in our study.^5^

In a previous study examining barriers to the use of capnography, interviews with physicians and nurses were not anonymous and were conducted by a nurse practitioner. The research team included a physician serving in the department of those interviewed, which may have biased the responses, owing to participants desiring to appear more accepting of capnography than they truly were.^14^ The results we obtained seem to correspond to findings in literature seeking to understand barriers to the implementation of certain practices and technology in medical settings. We identified inexperience, lack of knowledge, patient tolerance, lack of comfort with its use and lack of policy as significant barriers, similar to other studies.^14^ These obstacles coincide with a lack of cohesive attitudes, which were highlighted in our survey and could be attributed to the categories mentioned previously: education, culture, experience and elements specific to individual nurses.

### Educational intervention

The results of the survey might be indicative of an unclear definition of what exactly constitutes ‘nurse education’. Most staff interviewed after a 2015 trial using capnography requested additional education and training.^14^ Staff education has shown varying degrees of success in studies.^9 14^ Distinguishing between education and training could be a key to understanding nurse behaviour; this difference is meaningfully articulated as the contrast between competence and competency, the former being the knowledge and potential to perform, while the latter indicating the integration of knowledge.^17^ For successful team learning of technology, multiple iterations were seen as important to implementation of technology in a hospital setting^9^; repetition and reinforcement as effective teaching strategies should be given sufficient consideration when education efforts are organised.^18^

### Experience

Those with previous experience using capnography have been shown to demonstrate a higher comfort level with its use.^14^ Experiencing aggravations such as false alarms over time impacts the view nurses have of the benefits of monitoring; increasing alarm fatigue drives dismissal of alarms, with significant patient safety impact.^19^ Experience alone may not be a reliable indicator of integration of technology, but its inconsistency across organisations as a successful facilitator of change does not negate its powerful impact on diffusion of innovation.^20^

### Culture

Culture affects decision making in hospitals and is linked to nurse perceptions; staffing, design of the hospital floor, and physician–nurse communication are some of the cultural criteria, with workload being a primary concern.^21^ In response to a question about the effect capnography monitoring had on the ability to carry out duties, 8 nurses of 22 (36%) reported negative effects, while 7 (32%) reported positive ones. Nurses interviewed in a previous study did not feel an additional burden by using capnography.^14^ These findings are important for future decisions regarding investment in capnography implementation. Although nurses may not consciously count capnography monitoring as a burden, they could be unaware of the demand that one more work variable takes, possibly leading to avoidance of its use or compromising other duties or concentration on important patient information or procedures.

What is valued by the sectors within a hospital competes for adoption into the system.^22^ Whether a physician values capnography, for instance, can determine a nurse’s attitude about employing it.^7^ Hospital culture is largely set by the behaviours of leaders, as opposed to their policies or promoted interests, which stresses the need for leaders to model and champion capnography use.^7 23^ How the stated values of a hospital are actually carried out by leaders could have effects on nurse perceptions that call for further study. Nurses have expressed the importance of patient-centred care as a driving force of management^11^; framing capnography in light of its benefit to the patient can aid in the process of nurses’ identification with the practice, enhancing buy-in.^9 15^ Findings from our survey showed a high value for capnography monitoring for unstable patients (mean 4.33, SD 1.02), compared with those with a range of comorbidities (mean 3.86, SD 1.01), suggesting that nurses might be unaware of the potential for escalation, even in low-acuity patients.

Our study found that patient satisfaction was perceived as negatively affected by those on capnography monitors; 17 of 22 nurses (77%) rated its effect on patient satisfaction as somewhat to very negative. Fears of poor patient satisfaction ratings are a cultural issue facing hospitals in light of their connection to funding; these fears impact nurses in varying ways depending on the institution.^24^

### Belief systems, mindset, personality and cognitive style

External factors are not the only ones to determine nurse attitudes; belief systems, mindset, personality and cognitive processing styles are agents that affect perspectives of nurses.^12 13^

Professional values are formed in part by nurse experience, culture and education and are often uniform within a staff; however, the internalisation of constructs and personal guiding principles that also make up value systems are more distinct to each nurse. Nurses’ values are closely tied with their decision making and patient care, thus affecting rates of adherence to select practices.^25^ The priorities and values expressed in a study examining the complexity of acute care nurses followed identifiable patterns: the importance of maintaining patient safety, staying on schedule, appearing competent and efficient to coworkers and maintaining patient and family satisfaction, all of which could be perceived by nurses as being hindered by capnography use.^12^ Participating in nursing research and applying it to practice had less importance as a factor determining nurse priorities^25^; the fact that capnography is often introduced through clinical trials makes it a candidate for being undervalued at its introduction. Nurses in our study reported that patient cooperation with wearing the device was low; 13 of 22 nurses (59%) described cooperation as somewhat to very low, with only 3 (14%) describing it as somewhat high, and none characterising it as very high. A nurse may develop negative attitudes towards capnography if it is perceived as a source of conflict between the nurse and patient. The interdependent relationship between critical care nurses and their patients is of high value as a source of identity and job satisfaction for nurses^26^; any threat to that relationship would be expected to contribute to nurses’ motivation to adopt a practice.

Some employees possess a dispositional inclination to feel a resistance to change and to behave on those feelings.^27^ Knowing that some nurses, by nature, have a personality that is averse to change can shape the training used to introduce capnography.

We specifically sought to better assess communication style in this study. Nurses were asked if they were more likely to use direct or indirect communication. The usefulness of this feedback is its connection to psychological safety or confidence in that the fear of effects on reputation or job security might be higher for nurses whose communication does not bring about desired results due to its being too indirect or too brash. Being a direct communicator may cause nurses to feel more assured of maintaining a positive connection with their patients while employing a practice with a negative effect on patient satisfaction; if nurses are less likely to feel negatively about communicating with patients regarding the use of capnography, then their perception and utilisation of it could be expected to be higher.

Learning styles are a function of cognitive processing and vary among individuals. They influence the transfer of knowledge gained through training and are tied to motivation.^28^ In turn, motivation is a useful indicator of resistance to change in hospital environments.^13^

### Functions of buy-in

The benefit of understanding barriers to capnography acceptance through the lens of buy-in could be significant. Facilitators of buy-in of a new practice are classified into three groups that are associated with the categories that we established as affecting nurse perceptions of capnography: psychological meaningfulness (nurse value of patient concern and safety, as well as a sense of contribution); psychological safety (nurse cognitive inclinations); and psychological availability (culture creating confidence in resources).^15^ Besides these facilitators of buy-in, factors to enhance buy-in include appealing to employee engagement (how the nurse will benefit, directly or through patient benefit, coinciding with nurse values); trust (in the process itself and those implementing the process, both culture and education related); personal connection and consequences (strongly linked to nurse values); and sufficient time allowed for the initiative (related to culture as well as nurse values).^15^

The team learning process for successful implementers of technology in a qualitative field study made use of preparatory practice sessions, promotion of shared meaning and reflective processes to engage learners with the new practice.^9^ A study of organisational learning cites team structures, incentives, psychological safety and use of analytic tools as influences on learning in organisational environments.^20^ Increasing nurse involvement in the process of implementation positively impacts buy-in and implementation of new technologies.^29^ Many models and theories exist regarding technology implementation; our survey provides additional support that the introduction of capnography is consistent with the need for a simultaneous development of new skills, beliefs and routines.^9^ Integrating reminders into the education, culture and experience of nurses is effective in the pursuit of innovation and might be an important way to increase use of capnography monitoring.^12^

### Limitations

There are limitations to this study. Our sample size was small, and respondents were not randomised. All nurses working midweek shifts in several consecutive weeks were eligible for inclusion in the study; while random, this may have presented selection bias. The internal validity of our survey was low. We would contend that this supports the hypothesis that inconsistency exists in the knowledge and acceptance of capnography.

The disparity of feelings towards capnography monitoring can possibly be understood better when distinguishing between using a device, such as the cannula in our study, and the practice of gathering end-tidal carbon dioxide data through other means. Our survey did not ask about feelings towards the actual device employed for monitoring capnography, which was often described as obtrusive by several patients and nurses.

The cost of implementation should be considered when assessing the perceived value of a new technology. Our study did not measure perceptions of capnography considering cost, and research should incorporate the impact of cost on perceived benefit.

Our knowledge of what information was presented to each unit of nurses during their education on capnography monitoring was limited to the durable materials and presentations that were available for review and the reports nurse managers and educators gave us regarding the nature of emails or meetings involving the capnography study. On occasion, we had the opportunity to witness or participate in a limited number of meetings with nurse educators and nurse managers, at which time concerns and questions could be addressed, but not in a prescriptive or methodical manner that was complete consistent across floors and educators. Written materials explaining the algorithm used in the study were available but not systematically taught. Our efforts to minimise variation in education were carried out by meeting with nurse managers and nurse educators from the units participating in the capnography trial regularly. Onboarding was conducted by the manufacturer 2 years prior to the study’s commencement, during which time changes in staff occurred and some of the foundational knowledge built on during training for the study could have deteriorated. However, introductory meetings that outlined the protocol and rationale of the capnography monitoring study were held by leadership, so the information from the original training was reinforced. Nonetheless, the consistency of knowledge translation and encouragement of the practice varied among units.

## Conclusion

Organised education efforts to help bring about acceptance of a new practice may be insufficient to guarantee integration of the practice into patient care. Perceptions of capnography are made up of complex factors that vary between institutions and units. A better understanding of these factors may translate to more sustained implementation. Our findings invite further investigation into the nature of nurse education, barriers to implementing sustained organisational learning around new technologies and how non-education forces affect nurse perceptions.
