# Supplementary material for: Nursing attitudes towards continuous capnographic monitoring of floor patients
Source: BMJ Open Qual. 2018 Sep 15;7(3):e000416. doi: 10.1136/bmjoq-2018-000416 (PMC6144903; doi:10.1136/bmjoq-2018-000416)
Supplement: Supplementary file 1 [file bmjoq-2018-000416supp001.pdf]

Confidential

Nurse Capnography Survey

Record ID ______________________

	Very Negatively	Somewhat Negatively	Not at all	Somewhat Positively	Very Postively
How is your ability to carry out your duties affected by adding capnography monitoring to patient care?	○	○	○	○	○

	Very Negatively	Somewhat Negatively	Not at All	Somewhat Positively	Very Positively
How do you feel patient safety is being affected by capnography monitoring?	○	○	○	○	○

	None	A Few	Many
How many experiences have you had with patients who had escalations of care that might have been prevented using capnography?	○	○	○

	Very Negative	Somewhat Negative	Not at All	Somewhat Positive	Very Positive
What effect do you think capnography monitoring has on patient satisfaction?	○	○	○	○	○

	Very Low	Somewhat Low	Neither High nor Low	Somewhat High	Very High
What level of cooperation would you describe patients as having with wearing the capnography cannula?	○	○	○	○	○

	Pulse oximetry more necessary	Equal	Capnography more necessary
Which choice best describes your attitude towards the necessity of pulse oximetry monitoring compared to capnography?	○	○	○

	Very Indirect	Somewhat Indirect	Open to Feedback	Somewhat Direct	Very Direct

Please describe your style of patient communication: direct (telling patients protocols they will follow) or indirect (suggestion that they follow certain protocols)?	○	○	○	○	○

	At Extreme Risk	Somewhat more at Risk	Not Changed	Somewhat Improved	Greatly Improved
If capnography monitoring were removed today, do you think patient safety would be	○	○	○	○	○

Please indicated the degree to which you agree with following statements:

	Strongly Disagree	Somewhat Disagree	Neither Agree nor Disagree	Somewhat Agree	Strongly Agree
Capnography provides important feedback DURING surgery	○	○	○	○	○
Capnography provides important feeback up to 1 hour post-surgery	○	○	○	○	○
Capnography provides important feedback up to 8 hours post-surgey	○	○	○	○	○
Capnography provides important feedback up to 24 hours post-surgery	○	○	○	○	○
Capnography provides important feedback in the presence of certain comorbidites	○	○	○	○	○
Capnography provides important clinical data in the unstable patient	○	○	○	○	○

	Not at all urgent	Somewhat urgent	Extremely urgent
What level of urgency do you currently assign to an alarm for apnea?	○	○	○
What level of urgency do you currently assign to an alarm for a respiratory rate violation?	○	○	○
What level of urgency do you currently assign to an alarm for a heart rate violation?	○	○	○
What level of urgency do you currently assign to an alarm for an SpO2 violation?	○	○	○

Additional Comments ______________________________
